# A phase I study of bendamustine hydrochloride administered day 1+2 every 3 weeks in patients with solid tumours

**DOI:** 10.1038/sj.bjc.6603776

**Published:** 2007-05-08

**Authors:** M Rasschaert, D Schrijvers, J Van den Brande, J Dyck, J Bosmans, K Merkle, J B Vermorken

**Affiliations:** 1 Department of Medical Oncology, University Hospital Antwerp, Wilrijkstraat 10, 2650 Edegem, Belgium; 2 Department of Medical Oncology, ziekenhuis netwerk Antwerpen - Middelheim, Lindendreef 1B, 2020 Antwerpen, Belgium; 3 Department of Cardiology, University Hospital Antwerp, Wilrijkstraat 10, 2650 Edegem, Belgium; 4 Clinical Research, Ribosepharm GmbH, Munich, Germany

**Keywords:** bendamustine, day 1+2 schedule, pharmacokinetics, phase I

## Abstract

The aim of the study was to determine the maximum tolerated dose (MTD), the dose limiting toxicity (DLT), and the pharmacokinetic profile (P_k_) of bendamustine (BM) on a day 1 and 2 every 3 weeks schedule and to recommend a safe phase II dose for further testing. Patients with solid tumours beyond standard therapy were eligible. A 30-min intravenous infusion of BM was administered d1+d2 q 3 weeks. The starting dose was 120 mg m^−2^ per day and dose increments of 20 mg m^−2^ were used. Plasma and urine samples were analysed using validated high-performance liquid chromatography/fluorescence assays. Fifteen patients were enrolled. They received a median of two cycles (range 1–8). The MTD was reached at the fourth dose level. Thrombocytopaenia (grade 4) was dose limiting in two of three patients at 180 mg m^−2^. One patient also experienced febrile neutropaenia. Lymphocytopaenia (grade 4) was present in every patient. Nonhaematologic toxicity including cardiac toxicity was not dose limiting with this schedule. Mean plasma P_k_ values of BM were *t*_max_ 35 min, *t*_1/2_ 49.1 min, *V*_d_ 18.3 l m^−2^, and clearance 265 ml min^−1^ m^−2^. The mean total amount of BM and its metabolites recovered in the first micturition was 8.3% (range 2.7–26%). The MTD of BM in the present dose schedule was 180 mg m^−2^ on day 1+2. Thrombocytopaenia was dose limiting. The recommended dose for future phase II trials with this schedule is 160 mg m^−2^ per day.

The cytotoxic agent bendamustine (BM) hydrochloride (Cytosan®, Treanda®, Ribomustin®, IMET-3393; ZIMET-3393; 5-[bis(2-chloroethyl)amino]-1-methylbenzimidazolyl-2-butyric-acid) is a multifunctional alkylating agent with a purine-like ring system and a novel mechanism of action. Ozegowski and Krebs (1963) first synthesised it in 1963 in former Eastern Germany . Owing to a hydrochloride residue to the butyric acid side chain, BM is soluble in water (Gandhi, 2002). Its alkylating activity has been described in preclinical studies, where – at least in high dosages – it causes the formation of intrastrand and interstrand crosslinks between DNA bases (Gandhi, 2002). In comparison with other more commonly used alkylating agents, such as cyclophosphamide or phenylalanine mustard, more DNA double-strand breaks are formed when used in equitoxic dosages (Barmam Balfour and Goa, 2001). In addition, DNA damage produced by BM is supposed to be repaired via base-excision repair rather than the alkylguanine transferase mechanism (Niemeyer *et al*, 2004). This suggests a different mode of action, which was recently confirmed when gene expression profiling analysis identified a different gene profile activated by BM (Leoni *et al*, 2004; Niemeyer *et al*, 2004). It offers an explanation for the lack of cross-resistance with other alkylating agents, as observed in anthracycline-resistant breast cancer cell lines and cisplatin-resistant ovarian cancer cell lines (Strumberg *et al*, 1996). Treatment with BM also induces a concentration-dependent apoptosis as evidenced by changes in Bcl-2 and Bax expression profiles in chronic B-cell lymphocytic leukaemia (Konstantinov *et al*, 2002; Schwänen *et al*, 2002). Synergism was demonstrated when BM was combined with fludarabine (Chow *et al*, 2001). Further evaluation indicated that the synergistic effects were associated with the downregulation of inhibitors of apoptosis proteins, prostate-apoptosis-response-gene 4, and death-associated protein (Daxx) and with enforced caspase activation (Chow *et al*, 2003).

Preclinical studies indicated that BM is selectively taken up from the plasma in the liver where it undergoes extensive first pass metabolism involving conjugation with glutathione (Bezek *et al*, 1996; Teichert *et al*, 2005). Similar to other mustards containing the bis-chloroethyl moiety, two products of chemical hydrolysis, namely monohydroxy-bendamustine (OH-BM) and dihydroxy-bendamustine (Di-OH-BM), have been detected. The elimination of unchanged BM and these metabolites is primarily renal (Weber *et al*, 1991; Preiss *et al*, 1998; Teichert *et al*, 2003). However, the main biotransformation products N-dimethyl-BM and *γ*-OH-BM are excreted with the bile (Bezek *et al*, 1991; Preiss *et al*, 1998; Teichert *et al*, 2006). In preclinical studies, acute toxicity is observed in bone marrow and intestines, while the kidneys, testes, prostate, and the lymphatic tissues are prone to subacute toxicity (Horn *et al*, 1985).

After its preclinical development, BM was first tested in multiple myeloma in 1969 and went into clinical use from 1971 onwards. After Germany's reunification, BM was marketed in the whole country on the basis of a so-called ‘fictitious’ registration. As a result of a re-registration procedure in Germany, the first full registration was granted in 2005. Only recently the drug was re-discovered in the United States and a whole range of preclinical and clinical trials were initiated. Just now clinical trials are being started in Japan. Especially, its antilymphoma activity and the lack of complete cross-resistance with the other alkylators fuels ongoing scientific interest.

Previous phase II studies of monotherapy BM demonstrated impressive remission rates combined with good tolerability in relapsing and/or refractory non-Hodgkin's lymphoma (NHL) (Heider and Niederle, 2001; Bremer, 2002; Weidmann *et al*, 2002; Friedberg *et al*, 2004). Moreover, in combination with vincristine and prednisone (BOP), BM demonstrated higher 5-year survival rates (61 *vs* 46%) compared to cyclophosphamide containing standard regimen (Herold *et al*, 2006). Furthermore, the doublet BM/rituximab is evaluated in NHL because of its synergistic activity and high response rate (70%) in phase II trials (Kanekal *et al*, 2004; Rummel *et al*, 2005a, 2005b). Bendamustine is also actively investigated in multiple myeloma where the combination with prednisone proved to be more efficacious than the standard treatment: melphalan and prednisone (time to treatment failure: 14 *vs* 10 months, *P*=0.02) (Pönisch *et al*, 2006). In solid tumours, BM's lack of cross-resistance with other alkylating agents, its favourable toxicity profile, and the fact that it shows anticancer activity in second line and in the salvage setting in patients with pretreated metastatic breast cancer (MBC) (Höffken *et al*, 1998; Zulkowski *et al*, 2002), non-small cell lung cancer (NSCLC) (Reck *et al*, 1998), and small cell lung cancer (SCLC) (Schmittel *et al*, 2007) make it a valuable addition to the armamentarium of active anticancer drugs. Recently, a randomised phase III study compared cyclophosphamide, methotrexate, and 5-fluorouracil (CMF) with a comparable schedule in which cyclophosphamide was replaced by BM (BMF). The BMF schedule demonstrated superior progression-free survival in first-line MBC (8.2 *vs* 6.7 months for CMF; Von Minckwitz *et al*, 2005).

The earlier studies suggested a variety of schedules in which single agent BM could be used: that is, as a short intravenous infusion of 50–60 mg m^−2^ for 5 consecutive days every 4 weeks; 120 mg m^−2^ for 2 consecutive days every 3–4 weeks or 60 mg m^−2^ weekly. The most commonly observed toxicities were both haematologic (leukocytopaenia, thrombocytopaenia, lymphocytopaenia, and anaemia), and nonhaematologic, in particular gastrointestinal disturbances (nausea, vomiting, and mucositis) (Schrijvers and Vermorken, 2002). Some cardiac toxicity was also described, consisting primarily of intermittent tachycardia (Reck *et al*, 1998; Schöffski *et al*, 2000a, 2000b).

Only recently formal phase I testing resumed. These included a day 1+8 q 4 weeks schedule, a weekly schedule, a day 1 q 3 weeks schedule – all in solid tumour patients – and a day 1+2 q 3–4 weeks in B-cell CLL (Bergmann *et al* 2005; Lissitchkov *et al*, 2006; Rasschaert *et al*, 2007; Schöffski *et al*, 2000a, 2000b).

The present report summarises the toxicity and pharmacokinetic profile (P_k_) of BM with the latter schedule when used in patients with solid tumours with the aim to define maximum tolerated dose (MTD), dose-limiting toxicity (DLT) and to recommend more precisely a safe dose for future phase II studies with this schedule.

## PATIENTS AND METHODS

### Patient selection

Patients with histologically confirmed advanced cancer refractory to standard therapy or for which no standard therapy existed were eligible. Patients were adult individuals, legally competent, in a reasonably good general condition (World Health Organization (WHO) performance status 0–2), with a life expectancy of >3 months, and were included after obtaining informed consent. Those with disturbed liver function (aspartate (AST), alanine aminotransferases (ALT), and bilirubin >2 times the upper limit of normal values), disturbed renal function (serum creatinine >2 × ULN), inadequate bone marrow function (Hb ⩽8.0 g dl^−1^, WBC ⩽4.0 × 10^9^ l^−1^, platelets ⩽100 × 10^9^ l^−1^), inadequate cardiac function (left ventricular ejection fraction (LVEF) <50%), those with evidence of active infection or uncontrolled infection, epilepsy, or peptic ulcer and those with suspected central nervous system involvement were excluded from the study.

All patients provided signed informed consent. In accordance with the Declaration of Helsinki, International Conference on Harmonization - Good Clinical Practice (ICH-GCP) – guidelines and applicable local laws. The Ethics Committee of the University Hospital of Antwerp approved the protocol.

### Study design

This was a single-centre, open-label nonrandomised phase I trial to study the P_k_ and to define the safety and tolerability of BM hydrochloride administered by a day 1+2 every 3 weeks schedule.

Starting dose was 120 mg m^−2^ per day and dose increments of 20 mg m^−2^ per day were used, provided that no dose-limiting event occurred in the first cycle of the previous dose level. The study treatment was administered on an outpatient basis.

The individual treatment was continued until disease progression or unacceptable toxicity. Anti-emetic therapy was left to the discretion of the clinician treating the patient. The concomitant use of other cytotoxic or experimental agents was not permitted and haematopoietic growth factors (G-CSF, GM-CSF or erythropoietin) were not routinely given.

The study drug was supplied by Ribosepharm GmbH (Munich, Germany) in sterile vials containing 100 mg BM hydrochloride to be administered in 500 ml of 0.9% saline over 30 min as a peripheral or central intravenous infusion.

### Definition of DLT, MTD and schedule of dose escalation

Dose-limiting toxicity was defined as (1) any ⩾grade 3 nonhaematologic toxicity (except for alopecia and inadequately treated nausea or vomiting), (2) grade 4 anaemia or thrombocytopaenia, (3) leukopaenia (<1.5 × 10^9^ l^−1^) or thrombocytopaenia (<50 × 10^9^ l^−1^) for >14 days (lymphocytopaenia was not considered dose limiting), or (4) febrile neutropaenia.

Initially, three patients were to be included at each dose level. If no DLT developed, dose escalation would continue. If one of three patients developed a DLT, another three would be enrolled at that same dose level. If at least two of three patients or at least two of six patients developed an identical DLT, that dose level would be classified as the MTD. If no more than one of six patients developed DLT, dose escalation would proceed.

No intra-patient dose escalation was permitted. Once three patients completed the first cycle defined as 21 days after the first BM administration and had been observed for acute toxicity, patients were allowed to start treatment at the next dose level.

### Assessment of toxicity

Patients were assessable for safety analysis when at least one cycle was administered and when one observation was carried out afterwards. Monitoring of safety and tolerability was carried out by the National Cancer Institute of Canada, Common Toxicity Criteria (NCIC-CTC version December 1994). The evaluation of side effects was based on weekly outpatient visits with laboratory and clinical investigations, medical history, and a full physical examination. The following investigations were carried out before each cycle: toxicity assessment; physical examination, including heart rate, blood pressure, body temperature, and body weight; blood tests including blood sedimentation rate, coagulation parameters, differential blood count, serum analysis including sodium, potassium, calcium, creatinine, blood urea nitrogen (BUN), uric acid, AST and ALT, alkaline phosphatase, *γ*-glutamyl transpeptidase, lactate dehydrogenase, bilirubin, creatine kinase (CK), CK-MB, protein, albumin, c-reactive protein, glucose, and tumour markers. Urinalysis, urinary sediment, and creatinine clearance (calculated by Cockroft–Gault equation) were also evaluated.

Toxicity assessment, physical examination, pulse rate, differential blood count, and serum parameters (creatinine, BUN, AST, ALT, CK, CK-MB, and glucose) were repeated weekly.

For the assessment of potential cardiac toxicity, electrocardiograms (ECG) were performed on days 1 (before and after treatment administration), 2, 8, and 15 of each cycle. Left ventricular ejection fraction (LVEF) (evaluated by MUGA scan) was initially determined only before the first administration of BM. However, when we observed cardiac toxicity in another phase I study using a day 1 q 3 weeks schedule, which was running in our department at the same time (Rasschaert *et al*, 2007), the study was put ‘on hold’ after recruitment of six patients (three each in dose levels 120 and 140 mg m^−2^). Only after the other phase I trial was completed, the protocol was amended for evaluating the LVEF every other cycle and recruitment resumed. In addition, we assessed troponin-t, prothrombotic parameters (platelet function, aggregation tests (with ristocetine, collagen, epinephrine)) and clotting factors (eg, Von Willebrand antigen) in a subset of patients. Furthermore, the wall tracking system was used to assess flow-mediated and nitroglycerin-mediated vasodilation of the brachial artery as described previously (Pyke and Tschakovsky, 2005). These parameters determine endothelial function and by themselves have been correlated to exercise tolerance and coronary heart disease.

### Assessment of response

Response was assessed every other cycle. For this, WHO criteria were used: complete response, complete disappearance of tumour and signs, or symptoms of disease; partial response, >50% reduction in tumour (calculated as the product of the tumour's greatest diameter and its perpendicular measurement) for a minimum of 4 weeks; minimal response, 25–50% reduction in tumour for a minimum of 4 weeks; stable disease (SD), <25% reduction or <25% progression for a minimum of 4 weeks; and progressive disease (PD), >25% increase in tumour size.

### Pharmacokinetics

#### Plasma samples

Five millilitres (ml) venous whole blood was drawn on day 1, from the arm contralateral to the infusion arm into 7.5 ml monovettes containing lithium heparin at the following time points: 0 (predose baseline), 10, 20, 30 (end of infusion), 35, 40, 45, 50, 60, 75, 90, 105, 120, 180, 280, 360, and 480 min.

Immediately upon collection, the samples were transferred in ice water and spun in a cold (4–6°C) centrifuge at 2000 **g** for 4 min. The separated plasma was immediately deep-frozen at −70°C in three aliquots of approximately 0.8 ml in prelabelled 1.2 ml tubes (Nalgene, Nalge Nune International, Rochester, NY, USA).

#### Urine samples

Before the first treatment (on the day before or on the treatment day), a 2 ml predose urine sample was collected from each patient. The urine produced after starting the drug infusion (first micturition) was completely collected throughout cycles 1 and 2. Two 1 ml aliquots of each collection were stored at −70°C.

Plasma and urine samples were analysed using a validated high-performance liquid chromatography/fluorescence assay.

#### Pharmacokinetic evaluation

Pharmacokinetic calculations were performed by means of the pharmacokinetic software package WinNonlin Pro 4.0 (Pharsight Corporation, USA, 2002). Parameters were determined by non-compartmental analysis (NCA).

The NCA was based on a model requiring a constant infusion of the drug. The peak plasma concentration (*C*_max_) and the time to reach *C*_max_ (*t*_max_) were read directly from the concentration–time data. The area under the plasma concentration–time curve (AUC) was calculated by the trapezoidal method from the first to last measurable concentration and extrapolated to infinity (AUC_Inf_) using the ratio of the last measured concentration to the terminal slope. The latter was determined by log-linear regression analysis of the terminal phase. Clearance and volume of distribution (*V*_d_) were normalised to body surface area.

### Statistical analyses

Interpretation of the clinical data was by SPSS 11.5 statistical software (SPSS Inc., Chicago IL, USA); only descriptive statistics are given.

Results are presented in absolute numbers or as group medians with the range or mean values with standard deviation (s.d.) or standard error (s.e.) as indicated.

## RESULTS

### Patient characteristics

A total of 15 patients were enrolled in this phase I trial over an extended time period (September 2000 to December 2003). Their characteristics are summarised in Table 1. The median WHO performance status of these patients was 1 (range 0–2).

All patients had received prior treatment for metastatic or recurrent disease and were refractory to prior chemotherapy. The median number of prior chemotherapy regimens was 3 (range 1–5 regimens); in fact, 12 of the 15 patients had received 3 or more chemotherapeutic regimens. None of the patients had received BM before.

### Bendamustine administration

A total of 35 cycles were administered with a median of 2 cycles (range 1–8). Five patients received only two cycles; of the patients who received three or more cycles, one received four cycles, one six cycles and one eight cycles. All patients received the full planned dose and no dose reductions were needed.

Of the seven patients who stopped treatment after one cycle, three patients did not complete the first observation period of 21 days and underwent surgery for PD. Four patients stopped therapy after one cycle, and did not receive a second because of death owing to tumour progression (1), refusal (1), PD (1) and one patient stopped treatment because of dose-limiting haematologic toxicity.

Only two cycles were delayed. In one case (second cycle at 160 mg m^−2^) this was due to haematologic toxicity (thrombocytopaenia maximally grade 3 but no DLT). In the second case (sixth cycle at 160 mg m^−2^), this was carried out on request of the patient.

### Safety

The worst nonhaematologic and haematologic toxicities per patient experienced during the first cycle are shown in Table 2. No treatment-related deaths occurred.

#### Nonhaematologic toxicity

Nonhaematologic toxicity was generally mild and not dose-limiting. The most frequently encountered side effects were fatigue (13 patients – 87%), nausea (10 patients – 67%), loss of appetite (10 patients – 67%), and vomiting (8 patients – 53%).

One patient developed a grade 2 allergic reaction after the administration of BM at a dose of 140 mg m^−2^. He experienced shortness of breath with mild stridor, flushes, and tachycardia. All effects were reversible after drug administration was interrupted (day 1 of the first cycle of BM); the second dose (day 2) could be completed uneventfully following a slow infusion rate and the use of corticosteroids prophylactically. No alopecia or peripheral neuropathy was observed.

None of the eligible patients had clinically relevant coronary heart disease; however four patients entered the study with abnormal ECGs: one showed a right bundle branch block, the second a left anterior fascicular block, the third a first degree atrio-ventricular block, only one – the fourth showed non-specific T-wave abnormalities. Serial ECGs were performed according to protocol and showed cardiac toxicity. Sinus tachycardia (grade 1 NCI-CTC) was present in four patients (one patient at 120, one at 140, and two at 160 mg m^−2^). Premature supraventricular complexes were seen in one patient at 140 mg m^−2^ and premature atrial complexes and ventricular extrasystoles were observed in another patient at 160 mg m^−2^. No therapeutic intervention was needed. Furthermore, one patient (at 140 mg m^−2^) with a left anterior fascicular block before study entry developed ECG signs compatible with an infero-septal infarction on day 22 of the first cycle, at which time he was hospitalised because of gastro-intestinal bleeding and generalised malaise. Since no clinical symptoms of acute myocardial infarction or elevated CK/CK-MB were recorded, the ECG changes were evaluated as disease related (due to anaemia).

However, since ischaemic cardiac toxicity was observed in our day 1 every 3 weeks phase I study with the same drug, an analysis was performed in four patients at dose level 160 mg m^−2^ to identify any endothelial risk factors (see assessment of toxicity). No definitive relation between any of the tested parameters, use of the study drug and endothelial (dys-) function or cardiac ischaemia could be demonstrated. Furthermore, only one patient showed a decrease in LVEF (of 11%) after the second cycle. Unfortunately, none of the patients at the higher dose level (180 mg m^−2^) received more than one cycle; therefore no additional information on potential cardiotoxic effects at this higher dose could be obtained.

In conclusion, cardiac toxicity in this phase I study was present, but not considered as being dose limiting. All cases of sinus tachycardia were of mild/moderate severity and self-limiting.

#### Haematologic toxicity

Three patients presented with tumour-related anaemia (Hb <10 g dl^−1^) before treatment. Two of them received a blood transfusion and one used darbepoetine before the start of the first cycle (protocol violation). Eight patients developed anaemia (two at 120 mg m^−2^, one at 140 mg m^−2^, three at 160 mg m^−2^, and two at 180 mg m^−2^) for which blood transfusions were given in seven patients.

Myelosuppression, in particular thrombocytopaenia, was dose limiting (Table 2). Thrombocytopaenia grade 4 occurred in two patients at 180 mg m^−2^. It presented late during the first cycle (nadir on days 22 and 28, respectively) and was long-lasting (18 and 11 days, respectively). Both patients suffered from soft tissue sarcoma and had been extensively pretreated with alkylating agents; one had also received radiotherapy. One of these two patients also experienced febrile neutropaenia.

With two out of three patients at the 180 mg m^−2^ dose level having thrombocytopaenia grade 4, this dose level was defined as the MTD. The dose level of 160 mg m^−2^ was safely administered to six patients; the latter dose therefore was defined as the recommended dose for phase II studies.

Lymphocyte depletion (grade 4) was present in every patient on day 8 and at any dose level. Although lymphocyte depletion was long-lasting, no opportunistic infections were observed. However, six patients needed antibiotics for symptomatic infections (cystitis (2), streptococcal sepsis (1), acute bronchitis (1), fever of unknown origin (1), febrile neutropaenia (1)).

### Tumour response

No clinical or radiologic responses were observed in these patients. However, four patients had stable disease for variable periods of time.

One patient with a soft tissue sarcoma and one with renal cell cancer, treated at the 160 and 140 mg m^−2^ dose level, respectively, experienced stabilisation of disease during six and eight cycles. A third patient with renal cell cancer, treated at the 120 mg m^−2^ dose level, had stable disease after four cycles, but stopped therapy owing to a streptococcal sepsis. A fourth patient with colorectal cancer achieved a stable disease after two cycles at 160 mg m^−2^ but refused further therapy.

### Pharmacokinetic profile

The pharmacokinetic parameters of BM in plasma calculated by NCA are listed in Table 3. Plasma P_k_ data were available for only six patients while urine P_k_ data were available for five patients.

The mean elimination half-life of BM in plasma was 49.1 min, the volume of distribution 18.3 l m^−2^ and the clearance 265 ml min^−1^ m^−2^. These figures do not significantly differ from those observed in our day 1 q 3 weeks study (Rasschaert *et al*, 2007). Maximum plasma concentrations of BM were found at the end of the infusion. In the present study, all mean values of *t*_max_ and *t*_1/2_ estimated for the metabolites OH-BM, *γ*-OH-BM and *N*-dimethyl-BM were in the range of 35–64 min, and did not demonstrate a clear dose dependency.

Owing to low concentrations (below detection level) and interfering peaks, only limited P_k_s could be drawn for Di-OH-BM. Therefore, no complete pharmacokinetic evaluation was feasible for this metabolite.

### Urinary excretion of BM and its metabolites

About 93.5% of the amount excreted in urine was BM and its hydrolysis products, expressed as the sum of parent compound and all identified metabolites. The mean total amount of BM and its metabolites recovered in the first micturition was 8.3%, ranging from 2.7 to 26.0%, expressed as percentage of the administered dose.

## DISCUSSION

In this phase I study with BM hydrochloride given by a 30 min intravenous infusion for two consecutive days every 3 weeks, thrombocytopaenia grade 4 was the DLT at 180 mg m^−2^ per day. Other important toxicities were long-lasting lymphocytopaenia, observed from the first cycle onwards and present in every patient irrespective of the given dose, and some nonhaematologic toxicity, that is, fatigue, loss of appetite, nausea and vomiting.

The recommended dose for further phase II testing is 160 mg m^−2^ day. At this and lower doses, tachycardia was observed; however, this did not seem to be of clinical relevance. An analysis in a subset of four patients, at dose level 160 mg m^−2^, gave no evidence of platelet dysfunction or endothelial dysfunction, which could be attributed to the use of BM.

Overall toxicity has been quite similar in the different phase I studies performed with BM in patients with solid tumours. However, some schedule dependency has been noted.

In a day 1+8 q 3 weeks schedule, Schöffski *et al* (2000a) determined an MTD at 140 mg m^−2^, and reported fatigue and dry mouth as DLTs. They also observed a high incidence of lymphocytopaenia without opportunistic infections. Later the same investigators conducted a phase I study of weekly BM (Schöffski *et al*, 2000b) and reported an MTD of 80 mg m^−2^, with cholinergic events, fatigue and fever as DLTs. Again a near absolute lymphocytopaenia was noted (11 out of 12 patients). Flow cytometric studies demonstrated that BM had a deleterious effect on all lymphocyte subsets, but most prominently on B cells.

In a third phase I trial in which a single dose of BM every 3 weeks was studied, the MTD was determined at 280 mg m^−2^. The DLTs in this schedule were fatigue and cardiac toxicity (Rasschaert *et al*, 2007). At the MTD, ST segment and T-wave changes suggested ischaemic cardiac toxicity in three out of four patients and one patient experienced a QT prolongation. Non-specific T-wave changes and sinus tachycardia were seen at lower doses with that regimen.

The present phase I study allowed for a higher dose intensity (DI, 107 mg m^−2^ per week) at the recommended dose than found with other schedules used in phase I studies (Table 4) or those used in clinical practice so far. This observation is of importance since a concentration-dependent efficacy has been described in preclinical studies (Gandhi, 2002; Konstantinov *et al*, 2002; Schwänen *et al*, 2002).

The estimated mean values of BM's terminal half-live (*t*_1/2_) of 49.1 min and of its volume of distribution (*V*_d_) of 18.3 l m^−2^ are not different from those obtained in former pharmacokinetic evaluations (Matthias *et al*, 1995).

In the present study, all mean values of *t*_max_ and *t*_1/2_ estimated for the metabolites OH-BM, *γ*-OH-BM and *N*-dimethyl-BM were in the range of 35–64 min and no dose dependency was evident for these metabolites.

No valid evaluation could be performed for Di-OH-BM due to too few data. Bendamustine and the hydrolysis products monohydroxy and dihydroxy BM are open to chemical hydrolysis. Therefore, the high variability of pharmacokinetic parameters should be interpreted with caution.

This was particularly true for the urinary P_k_ data. As an example, the highly variable amount of BM determined in urine samples ranged from 0.8 to 50.2%, expressed as percentage of the sum of all compounds quantified in this study. Therefore, for evaluation, we decided to summarise amounts of BM and the two hydrolysis products (OH-BM and Di-OH-BM) assuming undesirable hydrolysis during sampling period and/or sample preparation before freezing. Hence, we calculated an amount excreted in urine of 5.2 and 1.6% for *γ*-OH-BM and *N*-dimethyl-BM, respectively, expressed as the sum of all compounds quantified in this study. These figures are in agreement with results observed in a previous P_k_ study (Teichert *et al*, 2006).

In conclusion, BM hydrochloride given on a day 1+2 q 3 weeks schedule has predictable haematologic DLT and acceptable nonhaematologic toxicities. Nevertheless, as in other phase I experiences, fatigue remains an unpleasant side effect also with this schedule. The recommended dose of 160 mg m^−2^ per day seems to be safe and allows for a relatively high-dose intensity in comparison with other schedules. However, considering the overall low number of treatment cycles per patient in this phase I study, additional information with this schedule in a more favourable patient group seems warranted.

## Figures and Tables

**Table 1:** Patient characteristics

	**Number of patients (*n*=15)**
*Gender*	
Male	8
Female	7
	
*Age*	
Median (years)	55
Range (years)	29–80
	
*WHO performance status*	
0	2
1	8
2	5
	
*Pretreatment*	
Immunotherapy	3
Chemotherapy	15
Surgery	14
Radiation	5
	
*Tumour type*	
Colorectal carcinoma	4
Soft tissue sarcoma	3
Renal cell cancer	2
Melanoma	2
Oesophageal cancer	1
Adrenocortical carcinoma	1
Osteosarcoma	1
Mesothelioma	1

**Table 2:** Incidence of selected adverse events: first cycle, all causalities

	**120 mg m^−2^ (*n*=3)**	**140 mg m^−2^ (*n*=3)**	**160 mg m^−2^ (*n*=6)**	**180 mg m^−2^ (*n*=3)**
**Grade (NCIC-CTC)**	**All/grade 3–4**	**All/grade 3–4**	**All/grade 3–4**	**All/grade 3–4**
*Blood/bone marrow*				
Haemoglobin	2/0	2/0	5/1	2/1
Platelets	1/0	2/0	3/0	3/2
Leukocytes	0/0	1/0	3/0	2/1
Neutrophils	0/0	0/0	1/0	1/1
Lymphocytes	3/3	3/3	6/6	3/3
Febrile Neutropaenia	0/0	0/0	0/0	1/1
				
*Constitutional symptoms*				
Fatigue	2/1	3/1	4/0	1/0
Allergy	0/0	0/0	0/0	1/0
				
*Gastrointestinal*				
Nausea	3/0	2/0	2/0	2/0
Vomiting	2/0	2/0	1/0	1/0
Aiorejia	1 /1	2/0	3/0	2/0
Constipation	0/0	1/0	0/0	1/0
				
*Cholinergic symptoms*				
Dry mouth	0/0	0/0	1/0	1/0
				
*Cardiovascular symptoms*				
Deep vein thrombosis	1/1	1/1	0/0	0/0
Tachycardia	1/0	1/0	2/0	0/0

**Table 3:** Individual pharmacokinetic parameters of bendamustine in plasma

**Subject**	**Dose (mg m^−2^ per day)**	**t_1/2_ (min)**	**t_max_ (min)**	**C_max_ (ng ml^−1^)**	**AUC_all_ (min ng ml^−1^)**	**AUC_Inf_ (min ng ml^−1^)**	**V_d_ (ml m^−2^)**	**Cl (ml min m^−2^)**
2	120	57.3	40	9011.5	490823.5	491145.7	24.3600	244.3
3	120	60.2	30	8907.3	540908.0	541672.9	19.2560	221.5
6	140	30.7	30	9978.4	437716.8	437991.4	14.1583	319.6
8	160	45.3	30	9966.1	448803.3	449333.0	23.2872	356.1
13	160	60.0	50	24474.6	1800220.3	1805225.4	7.6750	88.6
15	160	41.0	30	8797.7	444477.8	444631.4	21.2679	359.8
Mean		49.1	35.0				18.3341	265.0
s.d.		12.1	8.4				6.3460	103.6

Abbreviations: AUC_Inf_=AUC extrapolated to infinity; AUC_all_=area under the plasma concentration time curve from time of dosing to last observation; Cl=clearance; C_max_, maximum peak concentration; s.d.=standard deviation; t1/2=half life; t_max_, time to reach peak concentration; Vd=volume of distribution.

**Table 4:** Bendamustine in phase I (/II) studies

**Author**	**Phase**	***n***	**Bendamustine regimen**	**MTD/RD (mg m^−2^)**	**DI (mg m^−2^ week^−1^)**	**DLT**
Schöffski *et al*	I	19	d1, d8 q 4 weeks	160/140	80	Mouth dryness, fatigue
Schöffski *et al*	I	12	Weekly × 8	80/60	60	Mouth dryness, fatigue, fever
Rasschaert *et al*	I	23	d1 q 3 weeks	280/260	87	Fatigue, cardiac toxicity
Bergmann *et al* CLL	I/II	16	d1, d2 q 3–4 weeks	80/70	47–35	Granulocytopaenia, infection
Lissichkov *et al* CLL	I/II	15	d1, d2 q 3–4 weeks	110/100	50	Bilirubinaemia Diarrhoea
Present study	I	15	d1, d2 q3 weeks	180/160	107	Thrombocytopaenia
